# Impact of an unannounced standardized veteran program on access to community-based services for veterans experiencing homelessness

**DOI:** 10.1093/pubmed/fdab062

**Published:** 2021-04-30

**Authors:** Saul J Weiner, Alan Schwartz, Amy Binns-Calvey, Benjamin Kass, Timothy D Underwood, Vincent Kane

**Affiliations:** Center of Innovation for Complex Chronic Healthcare, Edward Hines, Jr. VA Hospital, Hines, IL 60141, USA; Jesse Brown VA Medical Center, Chicago, IL 60612, USA; Departments of Medicine and Pediatrics, College of Medicine, University of Illinois at Chicago, Chicago, IL 60612, USA; Departments of Medical Education and Pediatrics, College of Medicine, University of Illinois at Chicago, Chicago, IL 60612, USA; Center of Innovation for Complex Chronic Healthcare, Edward Hines, Jr. VA Hospital, Hines, IL 60141, USA; Departments of Medicine and Medical Education, College of Medicine, University of Illinois at Chicago, Chicago, IL 60612, USA; Center of Innovation for Complex Chronic Healthcare, Edward Hines, Jr. VA Hospital, Hines, IL 60141, USA; Department of Medical Education, College of Medicine, University of Illinois at Chicago, Chicago, IL 60612, USA; HopeOneSource, Arlington, VA 22201, USA; Wilmington VA Medical Center, Wilmington, DE 19805, USA

**Keywords:** health services, methods, poverty

## Abstract

**Background:**

The United States Department of Veterans Affairs established a program in which actors incognito portray veterans experiencing homelessness with pre-determined needs to identify barriers to access and services at community-based organizations.

**Methods:**

From 2017 to 2019, actors who varied in gender, skin color and age portrayed one of three scripts at all VA Community-Based Resource and Referral Centers (CRRCs) serving veterans experiencing homelessness in 30 cities and completed an evaluative survey. They carried authentic VA identification and were registered in a VA patient database for each identity. CRRCs were provided with reports annually and asked to implement corrective plans. Data from the survey were analysed for change over time.

**Results:**

Access to food, counselling, PTSD treatment, and hypertension/prediabetes care services increased significantly from 68–77% in year 2 to 83–97% in year 3 (each *P* < 0.05 adjusted for script present). A significant disparity in access for African American actors resolved following more uniform adherence to pre-existing policies.

**Conclusions:**

The ‘unannounced standardized veteran’ (USV) can identify previously unrecognized barriers to needed services and care. Audit and feedback programs based on direct covert observation with systematic data collection and rapid feedback may be an effective strategy for improving services to highly vulnerable populations.

Mystery (or ‘secret’) shoppers are employed by the retail and hospitality industries to assess customer service delivery in the private sector, and sometimes by government agencies to enforce federal law.^1–3^ A variant of the mystery shopper, utilized in health care research, is the unannounced standardized patient (USP), an actor trained to portray a medical condition and adhere to a script designed to collect data on health care delivery.^4^ They have been trained, for instance, to request medications that are not indicated because they ‘saw them on TV’, to complain of symptoms characteristic of an often missed diagnosis, and to drop clues they are struggling with psychosocial issues that are essential to effectively managing their care.^5^^,^^6^ Methodologists refer to USPs as ‘intrinsically risk adjusted’ because they enable apples-to-apples comparison of how different providers would take care of the ‘same’ patient.^7^

Research scientists in the Department of Veterans Affairs (VA) have employed USPs to study social and health services and refer to them as ‘unannounced standardized veterans’ (USVs).^5^^,^^8–11^ In addition to adhering to a script, USVs complete checklists and provide qualitative feedback designed to assess the quality and consistency of services as they are actually delivered. Because they are standardized, USVs are ideal for disseminating best practices across multiple sites charged with delivering the same services to a specific clientele.^7^

In 2017, the Veteran Health Administration (VHA) Homeless Programs Office (HPO) launched a USV quality improvement (QI) program to identify gaps and disseminate best practices in providing services to veterans experiencing homelessness. The program was established to evaluate 30 Community Resource and Referral Centers (CRRCs) nationally. CRRCs are located in strategically selected areas to provide both a refuge from the streets and a central location to engage veterans experiencing homelessness and at-risk veterans in services. Veterans are assessed and referred to appropriate health and mental health care resources, job development programs, housing options and other VA and non-VA benefits.^12^

The VA had invested heavily since the 1980s in programs serving veterans experiencing homelessness and, since 2009, embarked on an initiative to end veteran homelessness, spending over $1.2 billion annually on services.^13^^,^^14^ The effort has been fruitful with a nearly 50% decline in the last decade.^12^ Nevertheless, over 37 000 veterans still experience homelessness at any given time.^12^ The VA is committed to assure that the infrastructure in place to serve this population embraces a ‘No Wrong Door’ philosophy, so that when a veteran presents at any intake site, such as a CRRC, they are consistently afforded ready and prompt access into a suite of services with each encounter customized to meet their needs.^15^^,^^16^ A USV program offers the VA an opportunity to self-assess its responsiveness to its most vulnerable population and to utilize the information to modify practices and staff behavior as a learning organization.^17^

## Program Description

The QI program was created by an HPO working group, starting in 2016, composed of content experts including staff who had first-hand experience working on the front lines at CRRCs directly serving veterans experiencing homelessness. In consultation with a team at the University of Illinois at Chicago (UIC) that has extensive experience employing USVs in VHA, three scripts were developed, each a prototype of veterans requiring specific services:^18–20^ (i) an African American male Vietnam veteran with alcohol dependence; (ii) a Caucasian female Desert Storm veteran with untreated hypertension/prediabetes; and (iii) a male Operation Enduring Freedom (OEF) veteran with undiagnosed Post-Traumatic Stress Disorder (PTSD)—all seeking assistance with housing, food, benefits and employment. In rounds one and two, the actor portraying the OEF veteran was Caucasian, and in round three, the role was portrayed by two African American actors.

In addition to these scripts, a checklist was developed to be completed by USVs following each visit. It included measures of access to a range of services, customer services ratings, waiting time and number of steps, each scored and weighted. The checklist is included as an Appendix. Note that the VA utilizes the ICARE model to define and measure customer service. ICARE articulates the value and concomitant behaviors that apply across the entire organization.^21^ In addition to continuous measures, dichotomous items were included to identify correctable problems, such as unnecessary requirements to receive needed services, inaccurate information about how to access services and missing vital services. Each time a USV indicated that they experienced a barrier to receipt of a needed service, they also provided a written description of the barrier. Because follow-up after visits was also of interest, USVs provided cell phone numbers that could be monitored for voicemail or text by the research team. Actors were recruited and trained at the UIC Simulation and Integrative Learning (SAIL) Institute.

USVs were provided with valid VA photo identification cards to match the fake identities they portrayed. Matching identification information was entered into VA information systems so it could be accessed by CRRC staff during intake, and data were removed following encounters so as not to corrupt downstream data with false information. We followed a similar protocol that had been implemented for a study employing USVs several years earlier, updated for this project in partnership with several central VA offices.^5^

The working group agreed on several principles to guide the program. Foremost was that it would not be punitive. Programs designed to improve quality and reduce error are notoriously ineffective if they focus on individuals rather than systems.^22^ Hence, any personal identifiers would be redacted before data collected from sites were compiled into reports. At the same time, CRRCs were informed that they would be held accountable, meaning that they would get feedback on their performance from the national office that funds their services with the expectation that they address identified deficits and adopt practices found to be successful at other sites.

The program went live in 2017, with a USV visiting each CRRC annually between February and August. Since sites were located across the vast expanse of the United States, most site visits required plane travel. Prior to the initiation of the first round, all CRRCs were informed of the program as were VA leaders and union representatives through email notices and several national calls. Following the first round of visits, aggregate and site-specific findings were shared with each CRRC director. When barriers to access or other deficits were discovered, sites were asked to implement correctives based on information from sites that did not have similar deficits. The process was repeated between the second and third round. A new feature added to the program in the third round, in 2018–19, was rapid feedback to sites when significant barriers to access were experienced during visits. For instance, when a USV was turned away despite having the required documentation to obtain services, the HPO office was promptly notified and could then reach out to the site to alert them and institute immediate corrective measures.

## Methods

Using data on CRRCs from the QI program, we compared changes over the 3 year period 2017–19 in measures of access to needed services, customer service and the number of steps and amount of time from arrival at a CRRC to receipt of services. We also compared changes in reported barriers to services and changes in receipt of expected services. Finally, because of an observed difference in access to services for African American USVs compared to Caucasian USVs in the baseline year, we conducted a subgroup analysis of access based on skin color adjusted for case. The Veterans Affairs Central Institutional Review Board determined that the analysis of the HPO’s QI program did not constitute human subjects research.

## Results

Mean USV-scored measures of access, customer service (ICARE), process and time are provided, along with mean total, in Table 1, with 95% confidence intervals around the means. Although access and ICARE scores increased substantially from 2018 to 2019, the increment was not sufficient to achieve significance, given the small *n* (i.e. just 30 CRRCs in the national program). From 2017 to 2018, the process score significantly increased, indicating fewer steps to receipt of services, and the time score significantly decreased signifying longer time to receipt of services. Changes in reported barriers to needed services are documented in Table 2, showing significant improvements (decreases in barriers) from 2018 to 2019 for accessing food, counselling, referral for PTSD treatment services and for medical care for hypertension/pre-diabetes.

**Table 1:** Reported changes in access, customer service, number of steps, time and overall score over 3 years. Increases signify improvement

	2017 (*N* = 30)	2018 (*N* = 31)	2019 (*N* = 30)
Access score	279.2 (237.2, 321.1)	250.0 (201.6, 298.4)	319.2 (280.3, 358.1)
ICARE score	36.5 (31.8, 41.3)	37.1 (32.3, 42.0)	43.1 (40.0, 46.2)
Process score^a^	10.8 (9.2, 12.3)	12.5 (11.3, 13.7)	11.7 (10.4, 13.1)
Time spent^b^	42.5 (33.5, 51.5)	31.4 (24.8, 37.9)	34.2 (28.9, 39.5)
Overall score	369.0 (323.6, 414.3)	331.0 (277.3, 384.7)	408.2 (367.3, 449.1)

a 2017 vs. 2018, *P* < 0.05 adjusted for role portrayed by USV

b 2017 vs. 2018, *P* < 0.01 adjusted for role portrayed by USV

**Table 2:** Reported barriers to needed services over 3 years

	2017 (*N* = 30)	2018 (*N* = 31)	2019 (*N* = 30)
Housing access barrier	18 (60.0%)	13 (41.9%)	5 (16.7%)
Food access barrier^a^	12 (40.0%)	8 (25.8%)	1 (3.3%)
Employment/benefits counselling barrier^b^	14 (46.7%)	10 (32.3%)	4 (13.3%)
PTSD services barrier^a^	5 (16.7%)	7 (22.6%)	1 (3.3%)
Hypertension/pre-diabetes care barrier^a^	6 (20.0%)	8 (25.8%)	5 (16.7%)
Substance abuse services barrier	4 (13.3%)	6 (19.4%)	2 (6.7%)

Decreases signify improvement

a 2018 vs. 2018, *P* < 0.01 adjusted for role portrayed by USV

b 2018 vs. 2019, *P* < 0.05 adjusted for role portrayed by USV

Whereas Table 2 reports on barriers to needed services (decreases signify improvement), Table 3 reports on expected or desirable services (increases signify improvement). For instance, in the first row, the percentage of USVs reporting they were able to access service without going elsewhere first increased from 66.7% (2017) to 72.0% (2018) to 83.3% (2019). The same upward trend is seen for the second item (accessing services without having to be assertive) and for several others (e.g. accessing services without having to show military discharge papers—‘DD-214’). Again, as is the case for similar trends observed in Table 1, they were not substantial enough to achieve significance given the small *n* (i.e. 30–31 CRRC sites).

**Table 3:** Receipt of expected services over 3 years

	2017 (*N* = 30)	2018 (*N* = 31)	2019 (*N* = 30)
Access service without going elsewhere first	20 (66.7%)	18 (72.0%)	25 (83.3%)
Access without being assertive	19 (63.3%)	21 (70.0%)	27 (90.0%)
Provided transportation	22 (73.3%)	17 (54.8%)	20 (74.1%)
Easy to find	—	26 (83.9%)	28 (93.3%)
Screened for diversion^a^	—	19 (61.3%)	28 (93.3%)
Access without a DD-214^b^	20 (67%)	23 (76.7%)	27 (90.0%)
Access to tests	—	10 (76.9%)	11 (68.8%)
Ask for phone number or given phone	—	26 (83.9%)	29 (96.7%)
Bill of rights posted	—	—	18 (60.0%)
Bill of rights has contact info	—	—	15 (83.3%)
Received a follow-up call/text	15 (48%)	13 (43%)	12 (40.0%)

Increases signify improvement. A em dash indicates that the service was not measured in the corresponding year

a Refers to efforts by CRRC staff to divert veterans who they believe could find housing or other needs on their own or elsewhere

b Refers to DD Form 214, Certificate of Release or Discharge from Active Duty. Should not be required of Veterans carrying valid VA identification cards

**Fig. 1:**
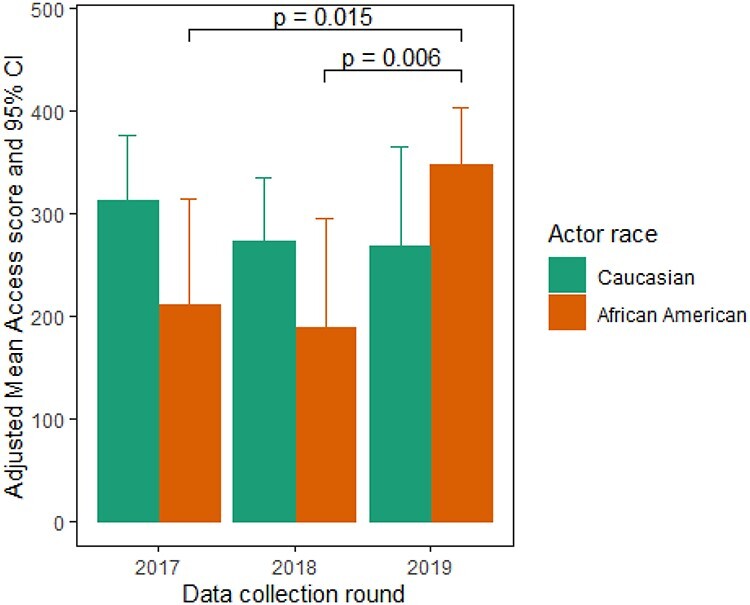
Changes in access for USVs portraying Caucasian and African American veterans.

In the first round of the program, in 2017, a marked discrepancy was noted between the access scores of the two USVs portrayed by Caucasian actors and the one portrayed by an African American actor. As noted in Fig. 1, the former had an adjusted mean access score 102 points (30%) higher than the latter. A similar pattern re-occurred in 2018, although the difference narrowed to 85 points. In 2019, a shift occurred with the access score for the African American portrayed USV visits rising above the Caucasian portrayed USV visit by 79 points. As noted, the changes in African American access were highly significant.

## Discussion

### Main finding of this study

The establishment of a USV quality improvement program has provided the Homeless Program Office with actionable information obtained by direct observation about how it is serving its clients. That information, in turn, has been utilized to institute changes in policy and practice, communicated to field-based service units, the CRRCs. In year 3 of the program, significant improvements have been documented in access to food, counselling, PTSD treatment referral and hypertension/prediabetes services, and a disparity in access for African American compared to Caucasian veterans when portrayed by USVs has resolved.

These changes all occurred in the third year. Although there were changes noted in the second year of the program (Table 1)—a decrease in the number of steps and an increase in time to complete the intake process—they are hard to interpret. We do not know, for instance, if USVs were spending more time in the waiting area or face-to-face with an intake worker collecting a detailed history and building rapport. Regardless, the changes did not persist into the third year. In contrast, the significant elimination of barriers to services (Table 2) documented from 2018 to 2019 is quite straightforward. The reasons, as reported by USVs, varied by site but are shown in aggregate in Table 3: for instance, CRRCs were more likely in 2019 than previously to provide services on site or, if not, to offer transportation, reducing barriers USVs experienced such as having to walk a long distance or find their own transportation to receive a service. While we can only speculate as to why it took 3 years to see substantial improvements, we surmise that CRRCs may have taken the feedback more seriously when they appreciated that it was not a one-off, meaning that if they did not make changes, they would hear about identified problems from senior leadership again.

The elimination of a disparity in access to services for the African American actors occurred after the USV program uncovered non-uniform application of VA policy regarding the documentation requirements to demonstrate eligibility for VA services—specifically with regard to the DD-214, which is not required (Table 3). As briefly noted above, the DD-214 is the Certificate of Release or Discharge from Active Duty that is issued upon a military service member’s discharge from active duty. Although it is one way to confirm veteran status, per VA policy it is not necessary to have on hand in order to receive veteran services as long as the individual is carrying a valid veteran photo ID. In the first round of USV visits, in 2017, the African American USV portraying a Vietnam veteran was frequently and incorrectly denied access because he did not have a DD-214. The Caucasian actors were rarely denied access because they did not have this document (data not shown). During the feedback process between rounds, the HPO reminded all CRRC sites, both in writing and verbally on national conference calls, that VA policy requires they accept a valid VA ID as sufficient evidence a client is a veteran. Following two rounds of feedback, the policy was consistently adopted resulting in few instances of USVs, Caucasian or African American, being turned away because they did not have their DD-214. This finding illustrates how a USV program can identify an inconsistency in the enforcement of a policy that resulted in a disparity in access, and how the information can enable an effective correction that addresses the disparity.

### What is already known on this topic?

There is an extensive literature on ‘street level bureaucracy’ that describes how front line workers in human service organizations, interfacing with ‘non-voluntary clients’ exercise wide discretion with relatively little accountability.^23^ The phenomenon has also been described among social workers, who are gatekeepers to publicly financed services.^24^ Weiner, who has studied front line discretion among low-wage intake staff in large safety net institutions, has observed that these individuals are often misinformed about policy, e.g. whether and when to approve services to patients lacking health insurance, further contributing to variation in accessible to essential service.^25^

These studies point to the capricious nature of social service delivery attributable to the variability in decision-making of front-line staff related both to their understanding and application of policy, and to their exercise of discretion in the absence of policy. The findings establish the need for a standardized process comparing service delivery based on direct, covert observation. A concern raised regarding such a process has been the element of deception. In the research context, deception is mitigated through an informed consent process in which the subjects voluntarily agree not to know when they are seeing a fake patient or client. In the context of a quality improvement program, such as the one reported on here, employees are similarly informed. Ethicists generally distinguish between deception protocols in which individuals are provided false information for the purposes of misleading them, and those in which they are fully informed about what is going to happen. USVs fall into the latter category, for which the threshold for demonstrating a benefit in excess of risk is lower.^26^

### What this study adds?

Our analysis illustrates the potential benefits of a program utilizing direct covert observation from the client’s perspective, with rapid actionable feedback, to improve services to a vulnerable population. A strength of USV data collection is that it uncovers hidden variations enabling rapid transfer of best practices from one site to another. For instance, at some CRRCs, USVs were told that they would have to first commute to a local VA medical centre to register and then return for further processing. Other sites simply had a direct phone line to the nearest facility so they could register without the additional step. As a result of the USV program, this ‘best practice’ was disseminated to all sites, with many adopting it, removing a barrier to service.

In addition, direct observation data collection methods, such as this USV approach, offer organizational leaders an authentic picture of what is happening on the front lines where services are actually delivered.^10^ For instance, when USVs were inappropriately turned away, there was no organizational record of their attempts to obtain service. The USV program was uniquely positioned to identify and rectify the problem. Many organizations rely on client surveys, which also do not capture such information. Another limitation of client surveys is that they are completed by individuals who do not typically have a reference for comparison. Unannounced standardized patients, upon whom our USVs are based, have been called ‘connoisseurs of care’ because they each conduct many visits, presenting with the same concern, and objectively collect the same data.^27^ This study suggests that USVs can significantly improve community-based services in programs where they are not currently utilized.

A fruitful area for future research on USV/USP type programs may be on social return on investment and cost effectiveness. While it was beyond the scope of this study to assess, such programs may be highly cost effective. The cost of the USV program was modest: about $114,000 United States Dollars (USD) per year to evaluate a federal program serving tens of thousands of veterans in 30 locations nationally. And there are economies of scale: the recent inclusion of another a larger VA program serving Veterans in which our USVs visited 70 sites, primarily in the same cities, increased the overall cost of the program by just $20 000 USD. Analytic models are available for measuring the value added of improving social service programs.^28^^,^^29^

### Limitations of this Study

There are several limitations to this study. First, because we conducted an analysis of just one program, it is difficult to generalize about whether the positive changes documented would occur in another setting. The recent addition of a second, larger, program, as noted above, may provide additional insight. Second, because this was an analysis of a QI program, not a randomized controlled trial, we did not have a control group against which to compare our findings. And third, it is possible that sites detected when they were visited by a USV and improved their performance selectively. We think this latter scenario is unlikely for several reasons: we did not send the same actors to the same sites in sequential years so as to avoid the risk of their being recognized; they presented with typical conditions indistinguishable from thousands of other clients; and they carried identification and were registered to be indistinguishable from real veterans.

## Disclaimer

The views expressed in this manuscript are those of the authors and do not necessarily reflect the position or policy of the Department of Veterans Affairs.

## Supplementary Material

Appendix_USV_Checklist_fdab062

## Notes

**Saul J. Weiner**, Deputy Director

**Alan Schwartz**, Acting Head

**Amy Binns-Calvey**, Research Assistant

**Benjamin Kass**, Research Assistant

**Timothy D. Underwood**, Executive Director of HopeOneSource

**Vincent Kane**, Director
