# Supplementary material for: Impact of an unannounced standardized veteran program on access to community-based services for veterans experiencing homelessness
Source: J Public Health (Oxf). 2021 Apr 30;44(1):207–13. doi: 10.1093/pubmed/fdab062 (PMC8904198; doi:10.1093/pubmed/fdab062)
Supplement: Appendix_USV_Checklist_fdab062 [file appendix_usv_checklist_fdab062.doc]

**CHECKLIST FOR Unannounced Standardized Veteran (USV)**

**ACCESS Items 1 of 2**

|  | **No Info Provided**  0 pts. | **General Information only. No specific person’s name and contact information provided (email, phone, and/or address).**  **You didn’t access service.**  25 pts. | **Cold Hand-off. Given information, along with a specific name and their direct email, phone and/or address who represents the service. You didn’t access service.**  50 pts | **Warm Hand-off. Given information, and a direct connection was made with someone on your behalf – on the phone, email, or in-person.**  **You didn’t access service.**  75 pts | **Accessed Service (e.g.: Shelter bed confirmed, provided food, and offered immediate primary or mental healthcare)**  100 pts | **Ex-perienced Barriers (Yes/**  **No)** | **Comments/**  **Description**  **(If a barrier was experienced, please describe)** |
| --- | --- | --- | --- | --- | --- | --- | --- |
| Were you provided with housing options that meet your needs? |  |  |  |  |  |  |  |
| Were you provided with food? |  |  |  |  |  |  |  |
| Were you provided with appropriate income counseling (employment, education, job-training, VA Benefits, SSI/SSDI)? |  |  |  |  |  |  |  |
| Did you receive Post Traumatic Stress Syndrome referral to a primary care or mental health provider (USV #1 only) |  |  |  |  |  |  |  |
| Did you receive information about accessing healthcare for high blood pressure/pre-diabetes? (USV #2 only) |  |  |  |  |  |  |  |
| Did you receive substance use disorder referral or assistance for your alcoholism? (USV #3 only) |  |  |  |  |  |  |  |

| Did you experience any barriers with housing? If so, please comment/describe. |  |
| --- | --- |
| Did you experience any barriers with food? If so, please comment/describe. |  |
| Did you experience any barriers with income counseling? If so, please comment/describe. |  |
| Did you experience any barriers with PTSD referral? If so, please comment/describe. USV 1 only. |  |
| Did you experience any barriers with health care info? If so, please comment/describe. USV 2 only. |  |
| Did you experience any barriers with substance abuse help? If so, please comment/describe. USV 2 only. |  |

**ACCESS Items 2 of 2**

|  | **No**  **0 pts** | **YES**  **25 pts.** | **N/A**  **0 pts** | **Comments** |
| --- | --- | --- | --- | --- |
| Was the site easy to find? (Signage was appropriate.) |  |  |  |  |
| Did you receive care without having  to go somewhere else first? (i.e. local VA Medical Center/CRRC) |  |  |  |  |
| Were you screened for diversion, to see if you could meet any of your own needs? (Encouraging you to identify immediate alternate housing arrangements like reconciling with former roommate.) |  |  |  |  |
| Upon showing your VA ID card, were you able to access services without a DD214? |  |  |  |  |
| If asked for medical tests (TB, STDs, Bed bugs, etc.) were you (a) able to receive those tests at the CRRC that day, or (b) provided same day transportation (shuttle, bus ticket, etc.) to get them elsewhere? |  |  |  |  |
| Were you able to access services without having to be very assertive?* |  |  |  |  |
| Were you provided transportation options to access some or all of the services (other than medical tests)? |  |  |  |  |
| Were you asked for a cell phone number and/or assisted with accessing a no cost Lifeline phone if you did not have one? |  |  |  |  |

*“Assertiveness” is defined as protesting or appealing when services are initially denied.

**If ever indicating “No”, please provide background with context:**

**ICARE Items:**

| **Please rate how the staff at the Community Resource and Referral Center met the following objectives:** | **Did**  **NOT**  **Meet**  **0 pts.** | **Semi-**  **Met**  **5 pts.** | **Meets**  **7 pts.** | **Exceeds**  **10 pts.** |
| --- | --- | --- | --- | --- |
| **Integrity –**  Did the staff act with high moral principles? Did they treat you in a professional manner? Did they maintain your trust and confidence? |  |  |  |  |
| **Commitment –**  Did the staff work diligently to serve you?  Were they organized? Did they appear to do their assigned job based on compliance or centered around your satisfaction as a customer? |  |  |  |  |
| **Advocacy –**  Did the staff identify your stated needs, consider them and advance your interests? |  |  |  |  |
| **Respect –**  Did the staff treat you with dignity and respect by listening to your story and calling you by your name? |  |  |  |  |
| **Excellence –**  Did the staff strive for your satisfaction? Were they willing to admit mistakes and work toward correcting them? |  |  |  |  |

| **Please notate any specific examples you want to report from the items above:** |
| --- |
|  |

**Process Steps Worksheet**

Please indicate the unique process steps that you were asked to complete and the time required completing them in minutes. Also, please include any comments you have for the steps.

The first few rows are some steps that are generally required, please indicate if they occurred by placing a “1” in the column. Then, in the rows below, please fill in the additional steps you were asked to complete.

| **Place a “1” if the step occurred** | **Unique Step Description** | **Time Spent**  **in**  **min.** |  | **Comments** |
| --- | --- | --- | --- | --- |
| *1* | *Sign in with registrar* | *5* |  | *No line* |
| *1* | *Waited in waiting room* | *120* |  | *Very messy – had to ask at desk if anyone was going to see me.* |
| *1* | *Interview with intake counselor* | *30* |  | *Counselor was rushed – insisted I needed to see someone else* |
|  |  |  |  |  |
|  |  |  |  |  |
|  |  |  |  |  |
|  |  |  |  |  |
|  |  |  |  |  |
|  |  |  |  |  |
|  |  |  |  |  |
| 3* |  | 155 |  |  |

**Summary**

| Total from ACCESS items |  |
| --- | --- |
| Total from ICARE items |  |
|  |  |
|  |  |
| **Overall Score** |  |

Additional Information for Background – Unscored

Date: _________________

Time Entered Facility: _________________

Time Exited Facility: _________________

Was the ID requested and validated? ________________

“Veterans’ Rights” sign was visible.

“Veterans’ Rights” sign contained a contact name and phone number.

Did the site share services with another organization? If yes, give the name of the organization.

Was any proof of homelessness required (e.g. “certificate of homelessness.)

Was the address you were given incorrect?

Were services unavailable the day you were there (e.g. job counselor not there)? If yes, what were those services?

Please indicate items you were told you needed for services:

DD214

State ID

SS Card

Birth Certificate

None

Other (please list)

Please describe if the DD214 was discussed and if it was a barrier to receiving services (please include if you were offered help getting your DD214.)

Any general comments on the visit (these comments will be shared with the site)?

Please write a narrative describing what happened during the day, especially anything not already

captured so far in this checklist. (This is shared with USV coordinator.)

**Follow up by site post-visit**

Type of contact:

Text:

Phone call:

Email:

Date of contact:

Any comments on the type of call/message received?
